# Beyond phylogeny: phytochemical diversity as a unique metric for biodiversity in the Gentianales

**DOI:** 10.1111/nph.70653

**Published:** 2025-10-21

**Authors:** Adam Richard‐Bollans, Eliot Jan‐Smith, Daniele Silvestro, Melanie‐Jayne R. Howes

**Affiliations:** ^1^ Royal Botanic Gardens, Kew Richmond TW9 3AE UK; ^2^ Department of Biosystems Science and Engineering ETH Zurich 4056 Basel Switzerland; ^3^ Gothenburg Global Biodiversity Centre, Department of Biological and Environmental Sciences University of Gothenburg 41319 Gothenburg Sweden; ^4^ Institute of Pharmaceutical Science, King's College London London SE1 9NH UK

**Keywords:** biodiversity, chemodiversity, Gentianales, phylogenetic diversity, phytochemistry, Python

## Abstract

- In contrast to phylogenetic diversity (PD), phytochemical diversity is not often utilised to measure biodiversity, although it forms an important aspect of biotic variation. The aim of this study was to explore phytochemical diversity across the flowering plant order Gentianales and the extent to which PD reliably represents phytochemical diversity.
- We developed the phytochempy package to facilitate the collection of phytochemical data, including a range of methods for calculating phytochemical diversity. To analyse patterns of phytochemical diversity within the Gentianales, species were grouped based on their native botanical regions. The phytochemical diversity and PD of these groups was then calculated to evaluate the similarities and differences among these measures.
- Given available presence data on phytochemicals in the Gentianales, we observed strong correlations between PD and the richness and disparity aspects of phytochemical diversity, with weak or no correlations with phytochemical evenness. We also found that, for measures of phytochemical diversity that incorporate evenness, the observed associations are driven by the confounding factor of species richness (SR).
- PD correlates with phytochemical richness and disparity in the Gentianales, but not with evenness, and SR confounds some of these relationships, showing that PD is not a reliable proxy for phytochemical evenness.

## Introduction

There is much interest in the analysis of phytochemical diversity of plant lineages, for example, to assess the evolution of chemodiversity in response to herbivory (Becerra *et al*., 2009; Wittmann & Bräutigam, 2025) or to accelerate searches for bioactive compounds (Foito & Stewart, 2018; Ghirga *et al*., 2021). Phytochemical diversity could also provide critical information for conservation prioritisation, as it represents unique functional traits that underpin ecosystem resilience and potential utilitarian value, which may not be captured by phylogenetic metrics alone. However, the concept of phytochemical diversity is often used in a loosely defined way, and phytochemistry research is instead often focused on providing insights into diversity by elucidating the structures or abundance of particular phytochemicals in particular groups of taxa (e.g. Berger & Schinnerl, 2019; Lee *et al*., 2020; Defossez *et al*., 2021; Shinyuy *et al*., 2025). Recent works, drawing on methods from measures of biodiversity in ecology, have begun to explore the quantification of phytochemical diversity and its various interpretations (Wetzel & Whitehead, 2020; Petrén *et al*., 2023; Sun *et al*., 2024; Thon *et al*., 2024). However, this work is often restricted to smaller groups of taxa, and as highlighted in Defossez *et al*. (2021), there is limited knowledge regarding phytochemical diversity across the plant kingdom as a whole.

In this work, we focus on the Gentianales, as the phytochemistry of this order has been studied extensively, and the phytochemical data are therefore relatively complete compared with other plant lineages. Gentianales is a large order of flowering plants (*c*. 23 000 species) comprised of five families: Apocynaceae, Gelsemiaceae, Gentianaceae, Loganiaceae and Rubiaceae, capturing roughly 6% of known vascular plant species. Various species and genera within this order are of economic importance, notably *Coffea* L., providing seeds for a vast global coffee industry (Torga & Spers, 2020). Numerous species provide pharmaceutical drugs, including *Cinchona* L., a source of the antimalarial quinine (Meshnick & Dobson, 2001), and *Catharanthus roseus* (L.) G.Don, a source of the anticancer drugs vincristine and vinblastine (Howes, 2018), as well as many species used in traditional remedies for various ailments (Milliken *et al*., 2021; Richard‐Bollans *et al*., 2023). Partly as a result of its ethnobotanical and economic importance, the phytochemistry of the Gentianales has been well studied, including, for example, numerous reports on the presence of alkaloids (Muhammad *et al*., 2003; Suksamrarn *et al*., 2003; Federici *et al*., 2009; Daley & Cordell, 2021; Jan‐Smith *et al*., 2025).

While the phytochemical properties of many species within this large plant lineage are well understood, the general relationship between phytochemical diversity and phylogenetic diversity (PD) remains unclear. PD reflects the accumulated evolutionary history of a group of taxa, which can be measured using branch lengths of phylogenetic trees (as in Faith's measure; Faith, 1992). Increased PD suggests increased time for which taxa have evolved independently and therefore is likely to produce greater diversity of traits (Crozier, 1997; Forest *et al*., 2007; Tucker *et al*., 2018). However, PD does not always provide a reliable indicator of different aspects of biodiversity (Cardillo, 2023).

Here, we analyse the relationship between phytochemical diversity and PD across biogeographic groups of species in the Gentianales. To this aim, we developed the phytochempy package, which facilitates the generation of phytochemical datasets and calculations of phytochemical diversity for large groups of taxa. We used this tool to calculate the phytochemical diversity of compounds found in botanical regions across the globe for species in the order. We compare the values of the different calculated phytochemical measures to highlight their similarities and differences. We hypothesise that phylogenetic and phytochemical diversity are strongly correlated but not equivalent, and that phytochemical diversity can provide a unique metric to measure biodiversity. However, following other work's finding that functional and PD is weakly or negatively correlated when species richness (SR) is accounted for Hähn *et al*. (2024), we also investigate how much of this association is in fact explained by SR rather than the accumulation of feature diversity during independent evolution of taxa. Finally, to highlight the potential differences in outcomes when biodiversity is measured using PD alone compared with phytochemical diversity, we identify regions of particularly high phytochemical diversity that may be overlooked when PD is prioritised.

## Materials and Methods

We developed the phytochempy (Richard‐Bollans, 2025b) python package to streamline the collection and compilation of large phytochemical datasets. Primarily, this package provides methods for downloading data on the presences of compounds in taxa from two large phytochemical databases: KNApSAcK (Afendi *et al*., 2012) and WikiData (Vrandečić & Krötzsch, 2014). Utilities are also provided for resolving plant names to the World Checklist of Vascular Plants (WCVP; Govaerts *et al*., 2021), enriching the compound data from a variety of sources and calculating chemodiversity for groups of compounds. Tutorials for these functionalities are provided with the package documentation.

### Compound data collection

Compound presence data were gathered from WikiData and KNApSAcK for taxa in the Gentianales. KNApSAcK is an extensive species‐metabolite dataset for plants that compiles metabolite–organism pairs from the published literature. WikiData is a vast community‐curated knowledgebase providing structured data across a broad range of topics. WikiData contains an extensive phytochemical dataset that is being actively improved through initiatives, such as the LOTUS project (Rutz *et al*., 2022). Names of taxa from these sources were resolved to accepted names in the WCVP v.12 using the wcvpy name resolution tool (Richard‐Bollans, 2025c). The collected data include primary metabolites (e.g. amino acids), which are essential for the normal functioning of a plant (e.g. for growth and development) and may be used in the biosynthesis of secondary metabolites, and secondary metabolites (e.g. alkaloids and terpenoids), which may have functional roles, such as for plant defence.

To uniquely identify compounds, SMILES strings (string representations of molecular structures) were used (Weininger, 1988). SMILES strings were standardised with the RDKit^†^ Python library sanitisation procedure and resolved to parent fragments, that is by removing any fragments (like salts or solvents) and returning the largest fragment. As different data sources use different standards for compound IDs, not all compound‐taxon pairs are provided with SMILES strings. Where CAS Registry IDs (CAS, 1978) were provided without SMILES strings, the CIRpy^‡^ Python package was used to interface with the NCI/CADD Chemical Identifier Resolver^§^ to convert CAS IDs to SMILES strings.

Compound data were also augmented with compound classifications based on NPClassifier (Kim *et al*., 2021) pathways, which groups them into fatty acids, polyketides, shikimates‐phenylpropanoids, terpenoids, alkaloids, amino acids/peptides and carbohydrates. These pathway enrichments were obtained via the GNPS API (Wang *et al*., 2016), and uncategorised compounds were removed.

To assess the diversity of natural groups of species and compounds, compounds were grouped based on the native distributions of species the compounds occur in, according to the WCVP and the World Geographical Scheme for Recording Plant Distributions (Level 3; Brummitt *et al*., 2001). When calculating phytochemical diversity of compounds in these groups, duplicate region–compound pairs were removed where compound uniqueness was determined by the standardised SMILES strings.

### Diversity metrics

When compiling data from existing data sources, the chemical profiles obtained, though extensive, are heavily reliant on the varying sampling effort. Certain species have been studied extensively for their phytochemistry due to their economic and cultural significance (e.g. *Coffea* L. species), and so the *measured* richness for such species is likely to be high compared with under‐explored species. This is an issue that is not restricted to chemical profiles, and in general calculating diversity in the context of varying sampling effort has been the subject of much discussion (Chao & Shen, 2003; Beck & Schwanghart, 2010; Chao & Jost, 2012; Roswell *et al*., 2021; Corre & Galy, 2023). Due to the novelty of measuring phytochemical diversity in the current context, we implement a variety of standard diversity metrics and corrected forms based on these discussions.

#### Compound diversity

The collected phytochemical data provide information on the reported occurrences of compounds, but not of their abundances in given samples or species. This simplification does avoid some issues relating to measurements of abundance (Wetzel & Whitehead, 2020), but limits the ability to calculate diversity metrics that rely on abundance; for example, Shannon's index (Shannon, 1948). We can, however, estimate ‘functional’ diversity based on the dissimilarities of identified compounds, similar to the approaches investigated in Mouchet *et al*. (2010); Petrén *et al*. (2023). Rao's quadratic entropy (Rao, 1982) is the most common such measure, and a simplified form without abundance is functional attribute diversity (FAD; Walker *et al*., 1999) defined as:

$$\mathrm{FAD}=\sum_{i=1}^{N}\sum_{j=1}^{N}d_{\mathrm{ij}}$$

where N is the number of compounds in the group and $d_{\mathrm{ij}}$ is a measure of dissimilarity between compounds i and j. The Taniomoto metric (Tanimoto, 1958), implemented in RDKit, is used in this study to calculate compound dissimilarity.

Related metrics that provide some correction for the sampling effort are modified functional attribute diversity (MFAD = FAD/*N*) (Schmera *et al*., 2009) and average pairwise distances (APWD = FAD/(*N* ^2^ − *N*)) (Heemsbergen *et al*., 2004). Each of these measures provide indications of the overall dissimilarities of compounds, with varying degrees of correction for the number of compounds and associated sampling effort. While functional Hill numbers (Chiu & Chao, 2014) have been increasingly popular distance‐based metrics, when being assigned the same abundance $\left(1/N\right)$ these metrics reduce to a function of FAD and are not included in this study. To summarise, FAD and MFAD measures the richness and disparity of compounds in a given group while APWD captures the average disparity of compounds in a group.

#### Pathway diversity

By contrast, rather than calculating diversity at the level of compounds, we may also consider the diversity of compound classes based on the biochemical pathways they originate from, similar to the usage of functional groups of plants in Tilman *et al*. (1997). Consolidating the compound data in this way allows for some approximations of relative abundance; that is, the abundance of a particular class can be calculated as the number of identified compounds from this class divided by the total number of identified compounds, and a similar approach is used in Becerra *et al*. (2009), González‐Medina *et al*. (2016) and Petrén *et al*. (2023). We calculated the Shannon index (H) (Shannon, 1948), using the relative abundances of each biosynthetic pathway from NPClassifier, that is:

$$H=-\sum_{i=1}^{P}p_{i}\ln p_{i}$$

where P is the number of different pathways and $p_{i}$ denotes the relative abundance of a given pathway in a taxonomic unit.

This measure considers the evenness of the different pathways as well as the associated richness (how many pathways are present), and so is likely to be influenced by the sampling effort, and so we implement other measures that provide some correction for this. Following Beck & Schwanghart (2010), we calculate the bias‐controlled Shannon's index (Hbc; Chao & Shen, 2003), which corrects for the sampling effort using an estimate of sample coverage:

$$\mathrm{Hbc}=-\sum_{i=1}^{P}\frac{p_{i}C\ln\left(p_{i}C\right)}{1-{\left(1-p_{i}C\right)}^{N}}$$

where N is the number of identified compounds and C denotes the sample coverage. The sample coverage for a collected sample of compounds is calculated using the number of singletons associated with the given pathways:

$$C=1-\frac{f_{1}}{N}$$

where $f_{1}$ denotes the number of pathways for which only a single compound from the pathway is found in the sample. Other methods for estimating coverage exist (Chao & Jost, 2012), for example, using doubletons, but the resulting metrics are very similar with our data.

Another related and popular measure of diversity that captures the likelihood that two random individuals in a group are of the same type is the Gini–Simpson index (G), calculated as 1 – Simpson's index (Simpson, 1949):

$$G=1-\sum_{i=1}^{P}p_{i}^{2}$$

Finally, to measure the evenness of the apparent pathways in a given group, we use Pielou's index (J) (Pielou, 1966), calculated as:

$$J=\frac{H}{H_{\max}}=\frac{H}{\ln P}$$

where P is the number of apparent distinct pathways in a given group. In the small number of cases where P=1, J is assigned null.

To summarise, each of the pathway‐based measures (H, Hbc and G) measures richness and evenness of the pathways found in a given group, while J measures evenness. Disparity between classes is not accounted for in any of these measures.

#### Rarefaction

As well as including diversity indices that correct in some way for sampling effort, we also use rarefaction as an alternative method to control for this. In the context of analysing the diversity of microbial communities, rarefaction has been shown to be a reliable way to control for uneven sampling effort (Schloss, 2024). For a given group of compounds, 1000 subsamples of size seven were taken and each diversity metric was calculated for each subsample. The rarefied versions of the diversity metrics were then taken to be the mean value across all subsamples. A subsample size of seven was used as this is equal to the number of possible NPclassifier pathways.

### Phylogeny

A Gentianales phylogeny for our analyses was built with the U.PhyloMaker R package (Jin & Qian, 2023) and used the GBOTB.extended.WP.tre megatree reported by Jin & Qian (2022), including the megatree of seed plants from Smith and Brown (2018). Species were added to the phylogenetic tree according to the World Checklist of Vascular Plants using the wcvpy python package. Given a group of species, PD was calculated using Faith's measure (Faith, 1992). Following Devictor *et al*. (2010) and Pardo *et al*. (2017), we calculated PD independent of SR by fitting regression models for SR against PD and using the residuals from the model. For this, we used a LOWESS regression model (Cleveland, 1979), which performs local linear fits and is robust to outliers. This was implemented with the *statsmodels* Python library (Seabold & Perktold, 2010).

### Analysis

Before analysis, groups of compounds were generated for each botanical region based on the species–compound data and native distributions of species. Groups with fewer than seven compounds were discarded as this is the minimum group size for the chosen rarefaction procedure. The diversity metrics described in Diversity metrics in the Materials and Methods section were calculated for each group, as well as the SR and PD. The resulting data were normalised using the Yeo–Johnson transformation (Yeo & Johnson, 2000), implemented in the scikit‐learn Python library (Pedregosa *et al*., 2011).

The similarities of the phytochemical diversity indices were assessed using Spearman rank‐order correlations (Spearman, 1904) and associated p‐values, implemented in scipy (Virtanen *et al*., 2020). Next, the associations between the phytochemical diversity indices and PD, SR and SR‐independent PD were assessed. Spearman correlations were used as we are interested in assessing the monotonicity of the relationships. All *P*‐values in this study were calculated using two‐sided tests.

To elucidate the potential differences in outcomes when biodiversity is measured using PD alone, compared with phytochemical diversity, we fit LOWESS regression models for PD against phytochemical diversity for each of the phytochemical diversity measures. To identify potentially under‐prioritised or over‐prioritised regions, we highlight those regions where the residual is greater or less than two SD from the mean residual value; that is where the phytochemical diversity is higher (or lower) than expected according to the PD.

## Results

### Collected data

Seventeen thousand three hundred and eighteen unique species–compound pairs were obtained for 1307 out of the 23 132 known species in the Gentianales. While this indicates some gaps in the data for further investigation, the global distribution of the species in the study provides a reasonable reflection of the distribution of all species in the order Supporting Information (Fig. S1).

After discarding regions with fewer than seven known compounds, we retained 340 out of a total of 369 botanical regions. On average, each of these regions is native to 37 of the species included in our study and is known to contain 502 unique compounds. Compound data for 147 of these regions contained every pathway grouping used in this study, with the regions containing six out of seven on average.

**Fig. 1:**
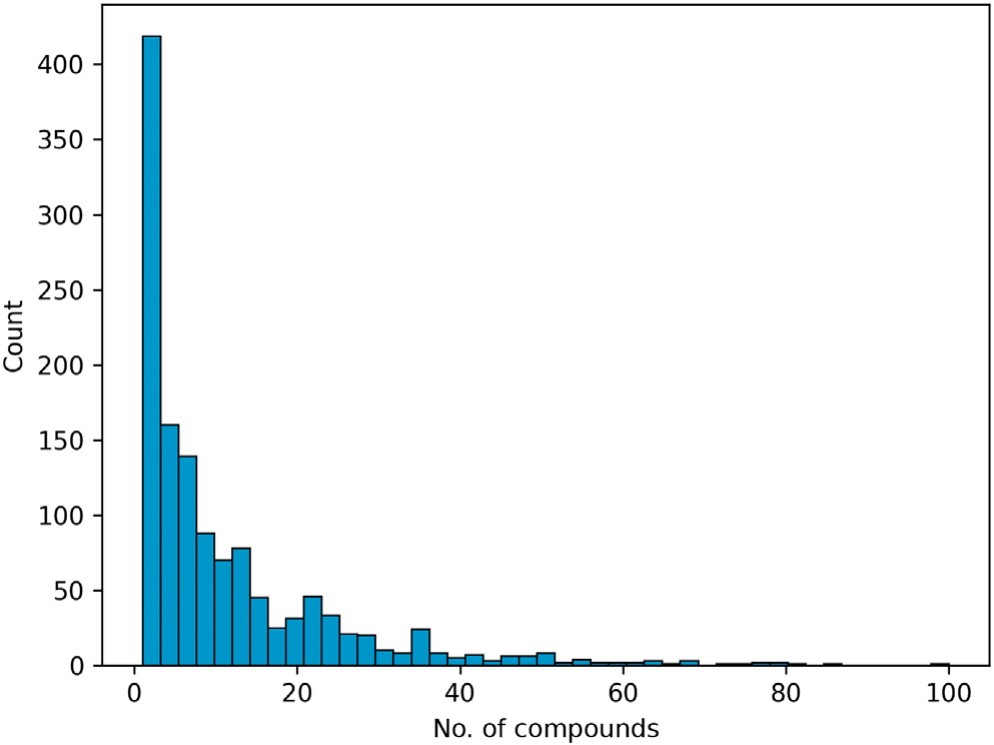
Histogram of the number of identified compounds per species in our study. For readability, this only shows counts for species with up to 100 identified compounds, but the full plot is included in Supporting Information Fig. S2.

**Fig. 2:**
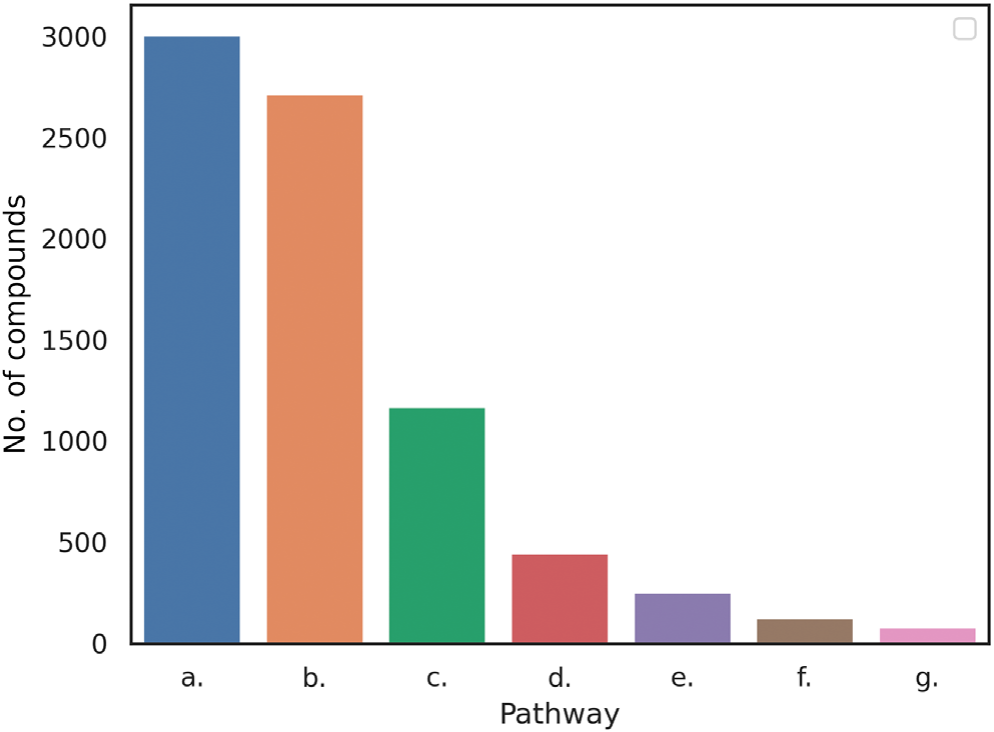
Bar chart showing the number of compounds in each pathway in our study. (a.) Terpenoids, (b.) alkaloids, (c.) shikimates and phenylpropanoids, (d.) polyketides, (e.) fatty acids, (f.) amino acids and peptides, (g.) and carbohydrates.

A histogram showing the distribution of the number of identified compounds per species is given in Fig. 1. This shows a clear skew in the data, with over 400 species with between one and three identified compounds. A small handful of species (including *Catharanthus roseus* (L.) G.Don and *Coffea arabica* L.) have over 100 identified compounds. A bar chart showing the number of compounds identified for each pathway is given in Fig. 2.

### Variation in phytochemical diversity measures

**Fig. 3:**
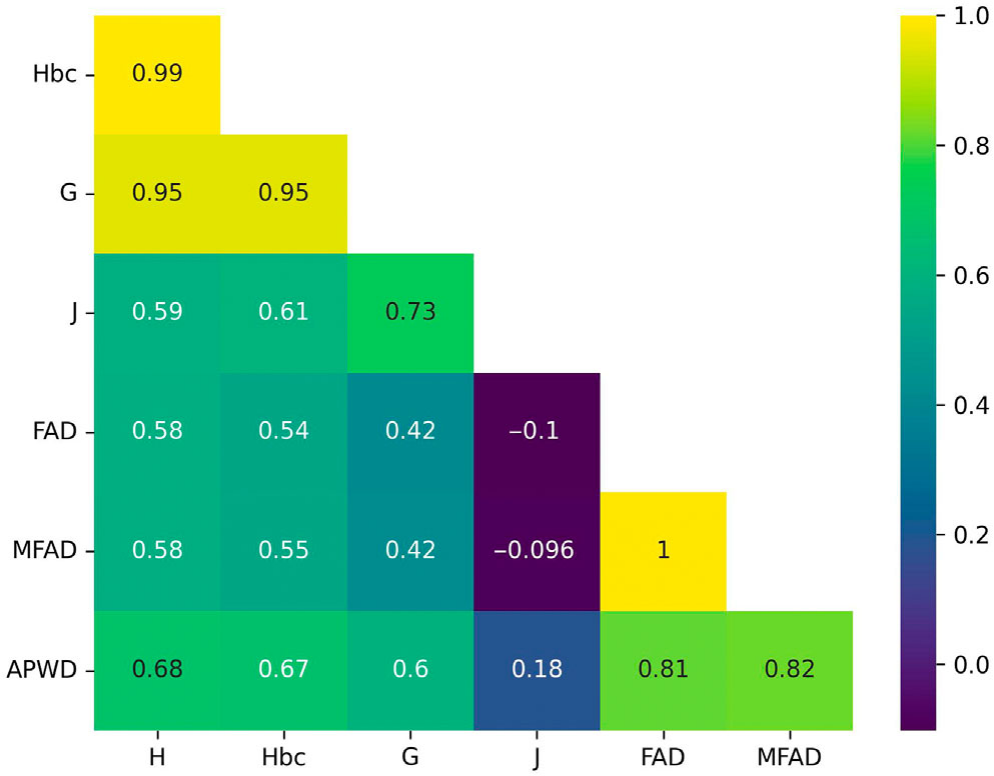
Spearman correlations of phytochemical diversity metrics comparing the Shannon index (H), bias‐controlled Shannon index (Hbc), Gini–Simpson index (G), Pielou's index (J), functional attribute diversity (FAD), modified functional attribute diversity (MFAD) and average pairwise distance (APWD).

Measures of the Spearman rank‐order correlation coefficients between each pair of diversity measures are provided in Fig. 3. We see that, with the exception of J, they are all strongly correlated and these correlations are also all significant in two‐sided tests (*P* < 0.0001). More detailed plots of the distributions are provided in Fig. S3.

Within the two groups of measures – (H, Hbc and G) and (FAD, MFAD and APWD) – there is very little variation and the measures are almost interchangeable for our datasets. In the case of J, there is a strong correlation with H, Hbc and G (*P* < 0.0001); however, it appears that groups with higher compound richness become less even (with respect to pathways) – for FAD and MFAD, respectively, ρ = −0.1 & −0.096 with *P* < 0.1 – while APWD appears to be weakly correlated with evenness (*P* < 0.001).

These observations are repeated in the case of the rarefied samples, with the exception that in this case J is significantly *positively* correlated to MFAD and FAD. We expect this is the case as the rarefaction process provides a similar control for sampling effort as APWD, and so MFAD and FAD using rarefied samples resemble APWD.

### Relationship to phylogenetic diversity

**Fig. 4:**
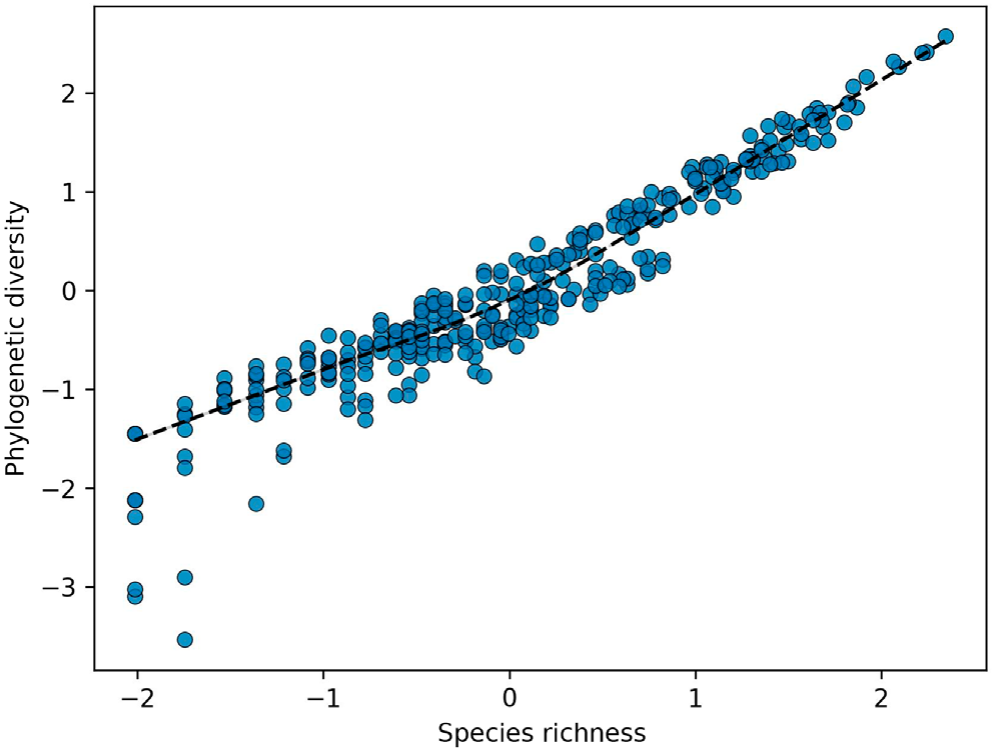
Species richness vs phylogenetic diversity (both Yeo–Johnson transformed), and the fitted LOWESS model. The plot shows the expected positive correlation between the number of species and the cumulative evolutionary time (phylogenetic branch length) separating them.

Figure 4 shows the relationship between SR and PD, along with the LOWESS regression method (*R*‐squared = 0.9), which we used to calculate SR‐independent PD.

**Fig. 5:**
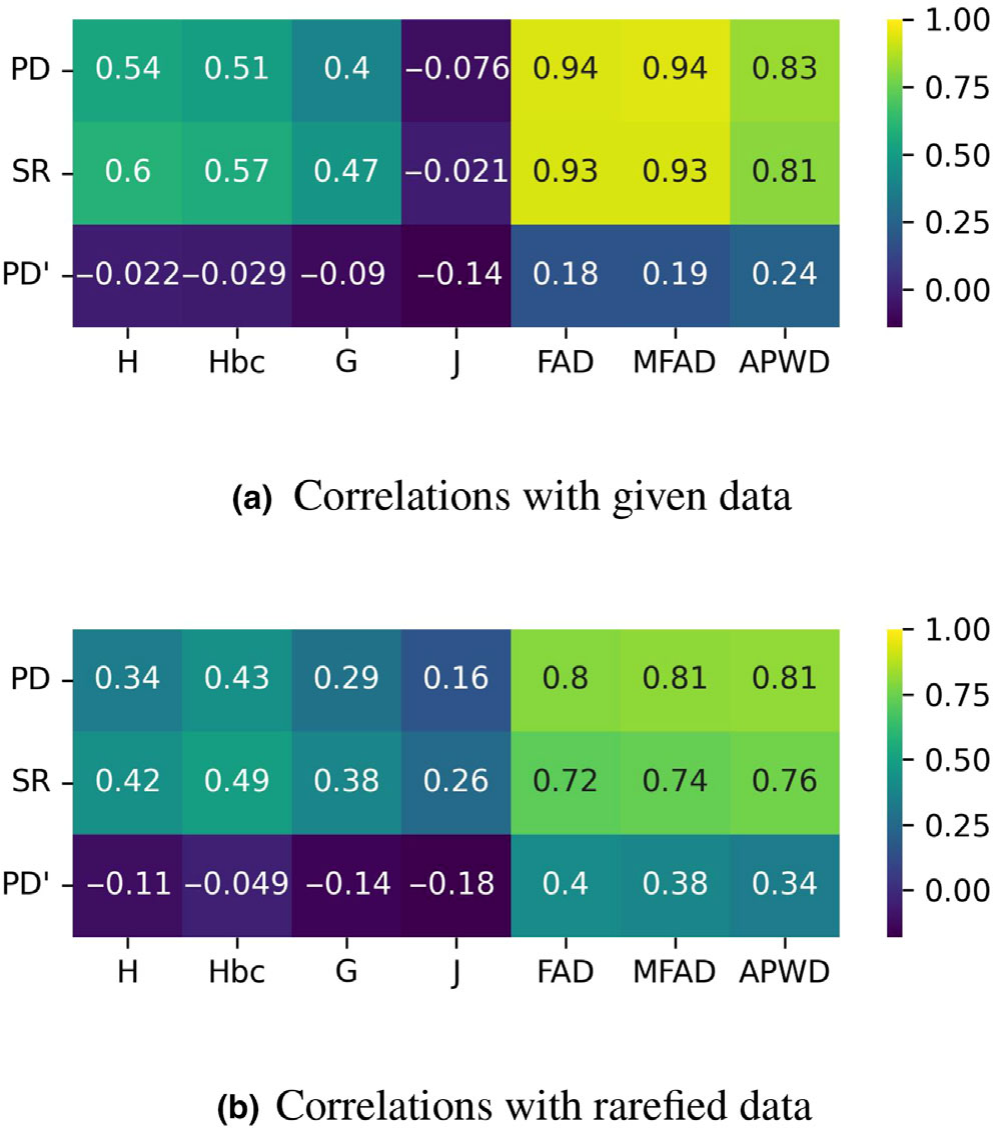
Spearman correlations between phytochemical diversity measures and phylogenetic diversity (PD), species richness (SR) and SR‐independent PD (PD').

The correlations of phytochemical diversity with phylogenetic diversity (PD), SR and SR‐independent PD (PD') are shown in Fig. 5. With the given data (a.), each diversity measure exhibits a significant positive correlation with both PD and SR (*P* < 0.0001), except for J (though not significant, *P* > 0.16). Again for the two groups of measures – (H, Hbc, G) and (FAD, MFAD, APWD) – we see two distinct patterns. H, Hbc and G have stronger correlations with SR than with PD, while FAD, MFAD and APWD have stronger correlations with PD than SR. These relationships are then reflected in the correlations when SR is accounted for with PD' – H, Hbc and G are negatively correlated with PD' while FAD, MFAD and APWD are positively correlated (only significant for FAD, MFAD and APWD *P* < 0.05). As with PD and SR, J is negatively correlated with PD' (*P* < 0.01). Using the rarefied samples (b.), these patterns are generally repeated, although J is now positively correlated with PD and SR.

While there is a very strong correspondence between PD and the measures that are strongly dependent on phytochemical richness (FAD and MFAD), we see a lessening of this effect when disparity (in the case of APWD) or evenness (in the case of H, Hbc and G) are considered, and, moreover, when only evenness is considered (J), we see mixed results when using the given vs rarefied data. These results indicate that PD may be useful as a general metric for capturing phytochemical diversity but does not provide a reliable measure of all aspects of phytochemical diversity.

Comparing the influence of PD and SR on phytochemical diversity for the phytochemical diversity measures that incorporate evenness; where there are positive associations between PD and phytochemical diversity, these are inverted when SR is accounted for; while for the measures that incorporate phytochemical disparity, correlations between both PD and PD' remain positive. These results highlight that for some aspects of phytochemical diversity, the associations between PD and phytochemical diversity are mostly driven by the confounding factor of SR, and that for these components of phytochemical diversity, SR may provide an improved, though still imperfect, measure of phytochemical diversity.

#### Outliers

**Fig. 6:**
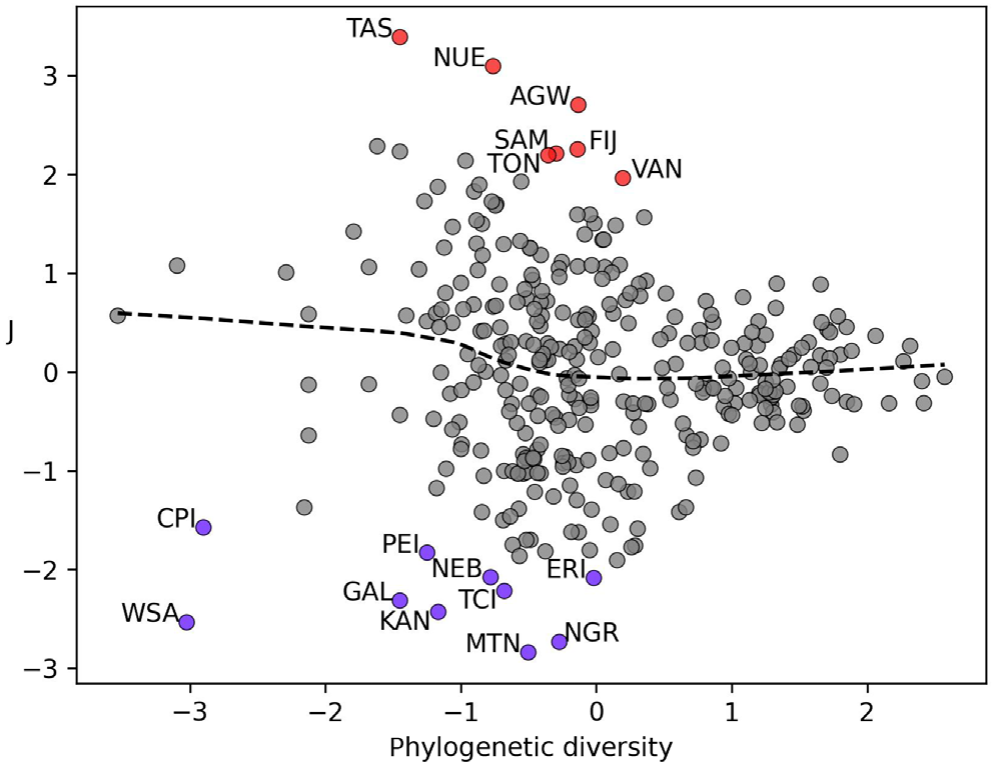
Visualisation of the relationship between phylogenetic diversity and J (phytochemical evenness). Outliers are highlighted in red and blue.

Although there are strong correlations between PD and phytochemical richness and disparity, we found that evenness is either uncorrelated or only weakly correlated with PD. It is clear that PD on its own poorly explains the variation in J, as seen in Fig. 6 (*R*‐squared = 0.009). Similar plots for the other phytochemical measures are provided in Fig. S4, but we focus here on J due to its lack of alignment with PD.

Seven regions identified with higher than expected J are Argentina Northwest (AGW), Fiji (FIJ), Niue (NUE), Samoa (SAM), Tasmania (TAS), Tonga (TON) and Vanuatu (VAN). Ten regions are identified as low outliers – Central American Pacific Islands (CPI), Eritrea (ERI), Galápagos (GAL), Kansas (KAN), Mauritania (MTN), Nebraska (NEB), Niger (NGR), Prince Edward Island (PEI), Turks and Caicos Islands (TCI), and Western Sahara (WSA). It is noteworthy that the highlighted high valued outlier regions, except AGW, are all islands or island nations, in contrast to the low valued outliers, given that islands are by far the places on Earth where the recent and ongoing extinction rates of plants and animals are highest (Gray, 2019; Rozzi *et al*., 2023). However, as with many geographic concepts (Varzi, 2001 and Gómez Álvarez *et al*., 2017), ‘island’ can be defined in a variety of ways, for example, by degree of isolation, size or distance from mainland as in Bastidas‐Urrutia *et al*. (2024). Currently, we highlight this as a potential avenue for further work but avoid a *post hoc* rationalisation and analysis of phytochemical diversity of islands.

## Discussion

Phytochemical diversity is an important aspect of the functional diversity of plants and the biodiversity of ecosystems. Our study focused on the phytochemical diversity of plant species in the Gentianales, aiming to provide insight into the relationship between phytochemical diversity and PD and the extent to which increasing PD increases phytochemical diversity. We developed the phytochempy package to facilitate the compilation of phytochemical data and the calculation of a variety of phytochemical diversity measures. Our study shows strong and significant correlations between PD and the richness and disparity aspects of phytochemical diversity, with weak or no correlations with phytochemical evenness. We also find that the positive relationships between H, Hbc and G and phytochemical diversity are mostly driven by the confounding factor of SR.

Our results show that PD may only provide a proxy for some aspects of phytochemical diversity, and choosing groups of species to maximise PD is not likely to maximise phytochemical diversity. While our study highlights the utility of PD in capturing some general trends, for researchers interested in preserving chemodiversity specifically, our work shows the importance of measuring this feature directly. Our findings align with the growing recognition of phytochemical diversity as a dynamic functional trait that directly mediates plant niche realisation processes, thereby shaping ecological interactions and ecosystem resilience (Müller & Junker, 2022).

### Utility of phytochempy

While we have focused on the interaction between phylogenetic and phytochemical diversity, there are many other avenues that could be explored with the application of phytochempy. For example, there is much interest in plant‐derived alkaloids due to their pharmacological and pharmaceutical importance (Cordell *et al*., 2001; Dey *et al*., 2020; Howes *et al*., 2020; Daley & Cordell, 2021). Phytochempy could be used to highlight alkaloid‐rich taxonomic units and botanical regions, which may have applications to aid natural product drug discovery. Using large‐scale phytochemical datasets could also help complement existing analyses of the evolutionary relationships of biosynthetic pathways, similar to Rønsted *et al*. (2012) and Maldonado *et al*. (2017) and Zhang *et al*. (2021), as well as extending chemotaxonomic work – the presenceof various classes of phytochemicals are already considered to be useful chemotaxonomic markers for a number of tribes and genera in the Gentianales (Jensen & Schripsema, 2001; Struwe, 2002; Mongrand *et al*., 2005; Endress *et al*., 2018; Berger & Schinnerl, 2019; Barny *et al*., 2021). Considering phytochemical diversity as a distinct trait, in contrast to the presence of specific compound classes, phytochempy provides functionalities to help explore this trait in more detail. By producing a diverse array of compounds, plants and fungi may increase their chances of producing useful compounds that benefit the organism (Jones & Firn, 1991; Weng, 2014; Sheldrake, 2021). It is an avenue for further work to assess whether phytochemical diversity in itself is an adaptive trait, that is if specific lineages existing under specific bioclimatic conditions are better adapted to produce more phytochemical diversity.

The chemodiv R package (Petrén *et al*., 2023) provides some similar functionality to phytochempy, with functions to query the NPClassifier tool and functions to calculate a variety of diversity measures. While there are differences in the implementations of some of these methods (e.g. we use local caching of NPClassifier results to improve large‐scale data mining; Petrén *et al*. include methods to calculate β‐diversity), the main differences arise from the data collection processes and associated data requirements, that is, we focus on retrieving data at scale where information on compound abundance is unavailable. While compound abundance is clearly an important factor, there are many ways an organism might modulate the concentration of a metabolite that it produces based on a/biotic conditions (e.g. Dumas *et al*., 2003; Qamar *et al*., 2024) but the binary presence in itself represents an important biosynthetic pathway and aspect of the phenotype.

### Sampling effort

As we compiled recorded data from existing data sources, we have little control over the sampling effort associated with each taxonomic group or group of compounds. Although the global distribution of species in our study appears to approximately resemble global patterns for species in the Gentianales, it is very likely that there are sampling biases as well as knowledge gaps in the data. As a result, we have aimed to incorporate methods that help to account for the sampling effort, for instance by limiting the effect of richness (as in the case of APWD and J), accounting for sample coverage (Hbc) and also running the analyses on rarefied samples. There are, however, many biases influencing the uses and studies of plants and associated data (Gaoue *et al*., 2017; Marks *et al*., 2023). For example, in the context of the data we have compiled, 315 unique compounds are known in *Coffea* L., likely due to its economic importance, compared with a mean of 55 per genus. It is also likely that certain plant parts are more commonly investigated for certain types of plants, which may influence the available data and calculated diversity. Similarly, some classes of compounds are more commonly investigated for certain types of plants, for example, peptides and amino acids for food plants, or fatty acids for plants whose seeds are of economic interest. Moreover, sampling effort in itself may not be evenly distributed. In Fig. S3, the relationship between the number of identified compounds in a region, N, and the other compound diversity metrics is observed. While a positive correlation is expected in most cases, as richness is one component of these measures, for example, APWD indicates that more sampling effort and phytochemical investigation are linked to more *diverse* sampling. For example, one study may focus on extracting alkaloids from a part of a specific plant (e.g. Yi *et al*., 2020), and a separate study may focus on a completely different compound class or plant part. More studies that are agnostic to compound classes are necessary to provide a more complete characterisation of phytochemical diversity.

Although the methods we have used help to mitigate issues with biases and knowledge gaps, the most reliable solution to this is to improve the quality of the available data. Recent initiatives have begun to improve on the compilation and dissemination of reliable phytochemical data (Rutz *et al*., 2022; Gomes *et al*., 2024); however, there is a lack of standardisation in the publishing of data on phytochemical occurrences. Many phytochemical publications use vernacular names for compounds without chemical identifiers, meaning that data on the discovered compound‐organism pairs are often difficult to integrate into easily accessible datasets.

### Relation to conservation

We have highlighted where phytochemical evenness diverges from PD, and specific regions that may be under‐prioritised with respect to preserving phytochemical diversity if we were to only consider PD when guiding conservation efforts. However, we do not currently identify these as ‘hotspots’ for three main reasons. First, our study is restricted to the order Gentianales. As this group captures roughly 6% of known vascular plant species, the phytochemistry patterns of this order might not be readily extrapolated to the full diversity of vascular plants. Second, as discussed above, there are likely effects of sampling biases and other data gaps. While we have accounted for these in some ways in this work, a more thorough investigation into these phenomena is required in order to fully correct for potential sampling biases. Finally, it may be the case that, while these regions have high phytochemical diversity, they may not be optimal for maximising *cumulative* phytochemical diversity. This idea is from Tietje *et al*. (2023) where they find that regions that maximise cumulative PD differ from the regions with high individual PD. Moreover, when considering outliers we have focused on J as this appears to be the measure least aligned with PD; however, specific metrics should be selected based on the particular aims of a given study depending on the importance of richness, disparity and evenness (Petrén *et al*., 2024) as well as the quality of the underlying data. Despite these limitations and nuances, phytochemical diversity can offer a complementary measure of biodiversity and should ideally be considered in setting conservation priorities.

## Competing interests

None declared.

## Author contributions

AR‐B contributed to the conceptualisation, software and data collection. AR‐B and DS contributed to the methodology. AR‐B, DS, EJ‐S and M‐JRH contributed to the interpretation, writing – original draft, and review and editing. All authors approved the final version of the manuscript.

## Disclaimer

The New Phytologist Foundation remains neutral with regard to jurisdictional claims in maps and in any institutional affiliations.

## Supporting information


**Fig. S1** The native distributions of species identified in the phytochemical data and species in the underlying population.
**Fig. S2** Histogram of number of identified compounds per species in our study.
**Fig. S3** Distributions of the phytochemical diversity metrics and their relationships.
**Fig. S4** Scatter plots of phylogenetic diversity against the phytochemical diversity metrics.
Please note: Wiley is not responsible for the content or functionality of any Supporting Information supplied by the authors. Any queries (other than missing material) should be directed to the *New Phytologist* Central Office.

## Data Availability

Collected data, scripts and analyses can be found on the GitHub repository (https://github.com/alrichardbollans/PhytoChemicalDiversity), which has also been archived through Zenodo (Richard‐Bollans, 2025a).

## Endnotes

^†^
RDKit: Open‐source cheminformatics. https://www.rdkit.org.

^‡^

https://github.com/mcs07/CIRpy.

^§^

https://cactus.nci.nih.gov/chemical/structure.
