# Supplementary material for: Beyond phylogeny: phytochemical diversity as a unique metric for biodiversity in the Gentianales
Source: New Phytol. 2025 Oct 21;248(6):3312–21. doi: 10.1111/nph.70653 (PMC12630460; doi:10.1111/nph.70653)
Supplement: Supplementary file 1 — Fig. S1 The native distributions of species identified in the phytochemical data and species in the underlying population. Fig. S2 Histogram of number of identified compounds per species in our study. Fig. S3 Distributions of the phytochemical diversity metrics and their relationships. Fig. S4 Scatter plots of phylogenetic diversity against the phytochemical diversity metrics. Please note: Wiley is not responsible for the content or functionality of any Supporting Information supplied by the authors. Any queries (other than missing material) should be directed to the New Phytologist Central Office. [file NPH-248-3312-s001.docx]

## *New Phytologist* Supporting Information

## Article title: Beyond Phylogeny: Phytochemical Diversity as a Unique Metric for Biodiversity in the Gentianales

Authors: Adam Richard-Bollans, Eliot Jan-Smith, Daniele Silvestro, Melanie-Jayne R. Howes

Article acceptance date: **01 September 2025**

The following Supporting Information is available for this article:

- **Fig. S1 The native distributions of species identified in the phytochemical data and species in the underlying population.**
- **Fig. S2 Histogram of number of identified compounds per species in our study.**
- **Fig. S3 Distributions of the phytochemical diversity metrics and their relationships.**
- **Fig. S4 Scatter plots of phylogenetic diversity against the phytochemical diversity metrics.**

**Fig. S1 The native distributions of (a) species identified in the phytochemical data and (b) species in the underlying population according to the World Checklist of Vascular Plants. Counts indicate the number of species native to each region.**
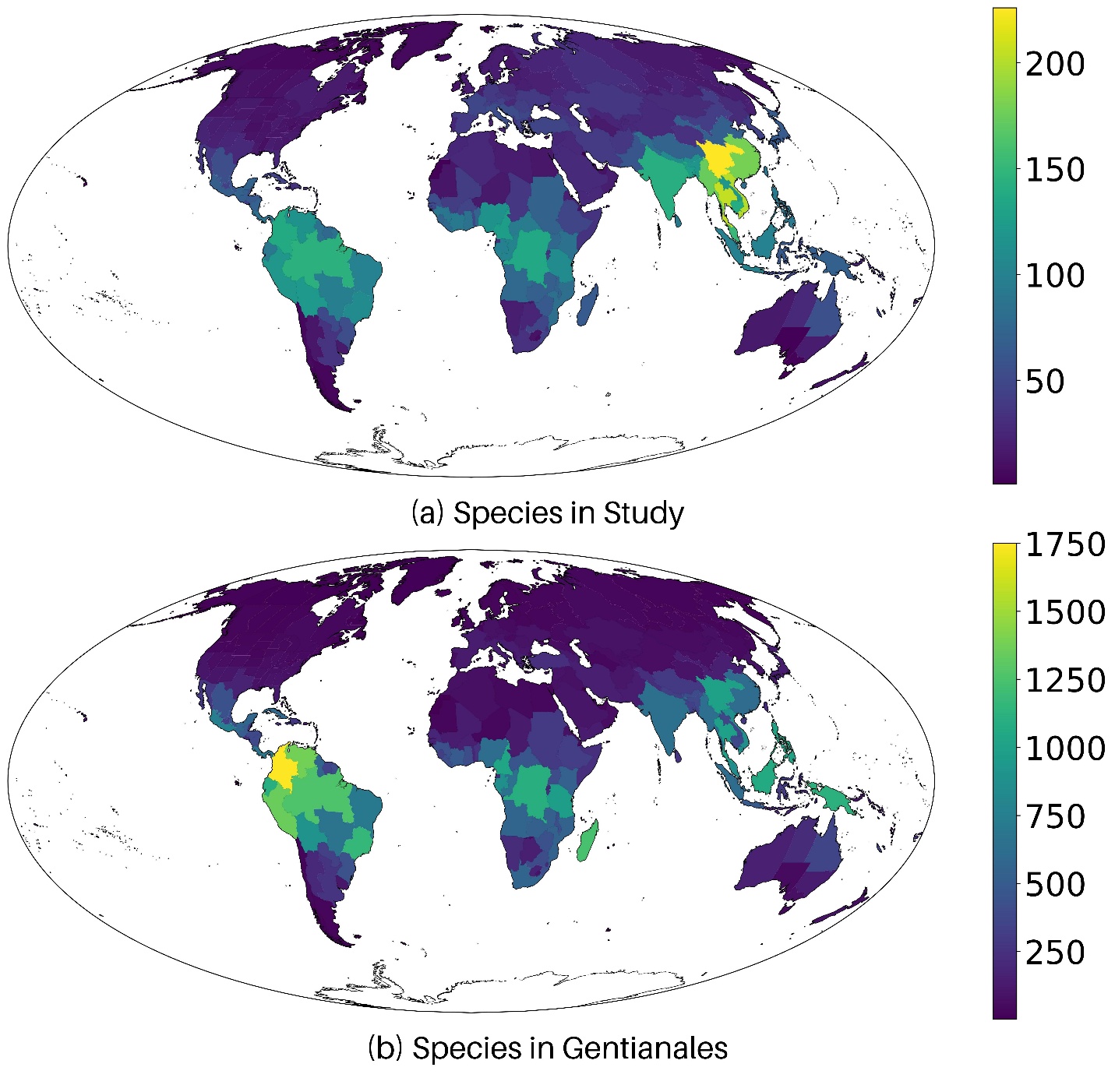


**Fig. S2 Histogram of the number of identified compounds per species in our study. Of the 1,307 species in the study, 19 species have over 100 identified compounds and are not included in the associated plot in the main article. *Catharanthus roseus* (L.) G.Don has the most identified compounds (347) .**


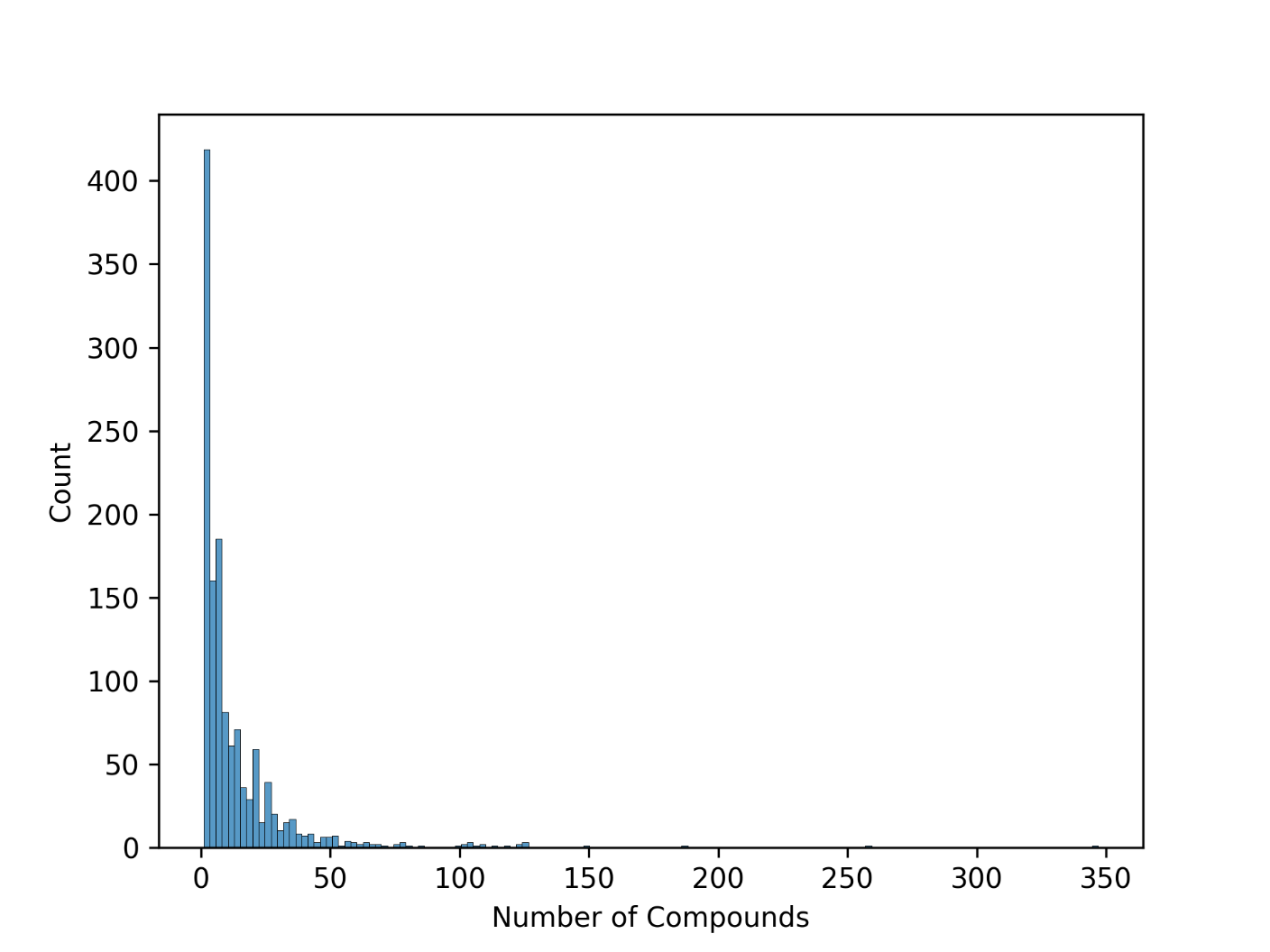


**Fig. S3 Distributions of the phytochemical diversity metrics and their relationships, generated with the seaborn pairplot function. In general, strong positive correlations between the number of identified compounds, N, and the phytochemical diversity metrics can be seen, particularly for FAD and MFAD.**


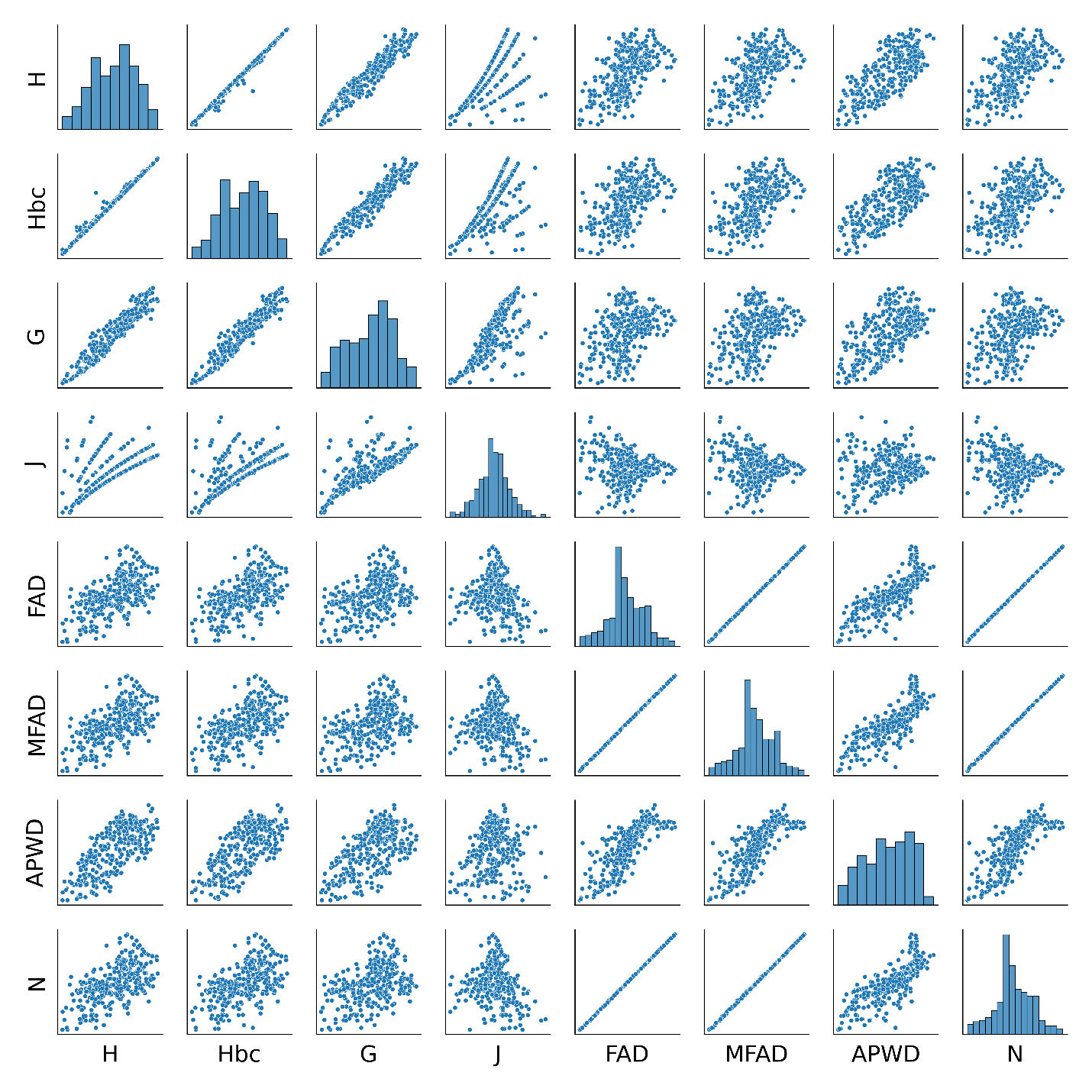


**Fig. S4 Scatter plots of phylogenetic diversity against phytochemical diversity metrics, including the associated LOWESS regressions. (a.) with given data and (b.) with rarefied data.**

| 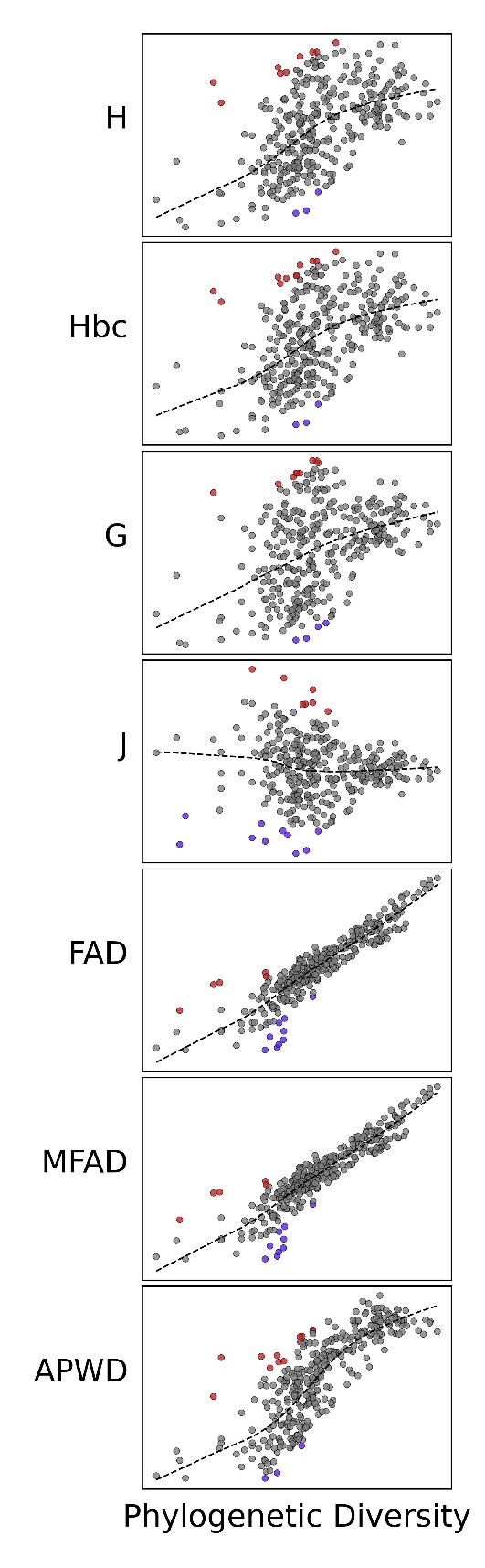 | 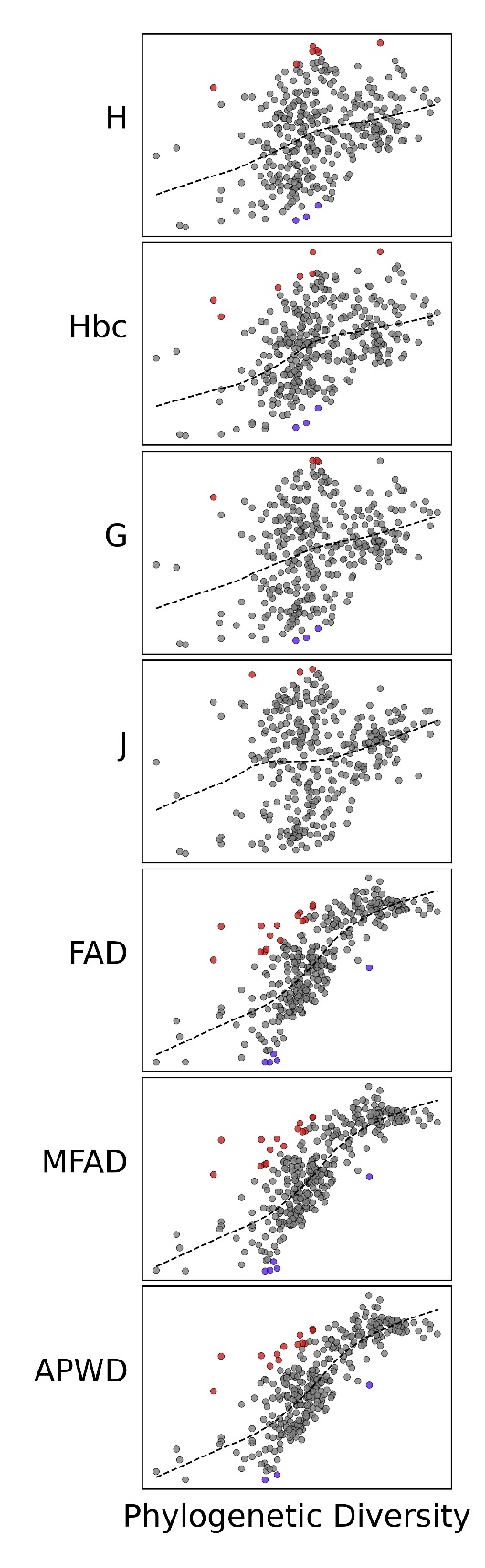 |
| --- | --- |
| **a.** | **b.** |
